# Folic Acid Ameliorates the Chronic Constriction Injury of Sciatic Nerve -Induced Memory Impairments in Rats

**DOI:** 10.5812/ijpr-166629

**Published:** 2026-04-27

**Authors:** Arunachalam Muthuraman, Mohamed Meeran Sheik Davooth, Seema Mehdi, Nallupillai Paramakrishnan, Satbir Kaur, Thiagharajan Venkata Rathina Kumar, Khian Giap Lim, Yamunna Paramaswaran

**Affiliations:** 1 Faculty of Pharmacy, AIMST University, Bedong, Malaysia; 2 Velammal Medical College and Research Institute, Madurai, India; 3 JSS College of Pharmacy, JSS Academy of Higher Education and Research, Mysuru, India; 4 Department of Pharmacognosy, Government Pharmacy College, Sikkim University, Sikkim, India; 5 Department of Pharmacology, Swami Vivekanand College of Pharmacy, Punjab, India; 6 Department of Pharmacognosy, College of Pharmacy, Madurai Medical College, Madurai, India

**Keywords:** Acetylcholinesterase, Nonverbal Learning Disability, Postoperative Cognitive Complications, Folic Acid, Memory Disorder, Sciatic Neuropathy

## Abstract

**Background and Objective:**

The present study investigated the effect of folic acid in a rat model of sciatic nerve injury (SNI)-induced cognitive dysfunction.

**Methods:**

In this study, six groups with six male Wistar rats in each group were used. The simple randomization method was adopted for unbiased assignment of animals based on age, sex, and weight variations. Folic acid (10 and 20 mg/kg) and donepezil (1 mg/kg) were administered by the oral (p.o.) route for 10 days. The rats' cognition was assessed by the Morris water maze (MWM) test. The assessment of the learning trial (acquisition) was the escape latency time (ELT), while the assessment of memory retention (retrieval) was the time spent in the target quadrant (TSTQ), which was measured in the MWM test. The brain samples of rats were used to assess biomarkers such as total protein, reduced glutathione (GSH), thiobarbituric acid reactive substances (TBARS), and acetylcholinesterase (AChE) activity. The behavioral data were statistically analyzed by a two-way analysis of variance (ANOVA) test, and biomarkers were analyzed by one-way ANOVA. The 95% confidence level (P < 0.05) was fixed for confirmation of statistical significance.

**Results:**

The administration of folic acid statistically (P < 0.05) reduced the SNI-induced elevated ELT and TSTQ levels compared to the sham control group. However, folic acid also prevented the rise in TBARS and AChE activity and the drop in GSH after SNI. The comparable outcomes were statistically (P < 0.05) similar to those of the donepezil-administered group.

**Conclusions:**

Folic acid has great potential to be used in treating peripheral nerve injury-associated cognitive dysfunction.

## 1. Background

Globally, more than 46 million people live with memory disorders and dementia. The prevalence is expected to escalate to about 132 million by the year 2050 (1). The major etiological factors of memory disorders are due to alterations in neuronal signaling, such as oxidative stress and neuroinflammation (2). Aging, diabetes, stroke, hyperhomocysteinemia, hypercholesterolemia, genetic factors, and peripheral nerve injuries have been shown to contribute to cognitive impairments (3, 4).

Peripheral nerve injuries are common and affect more than 100 in every 100,000 people annually due to accidents, violence, surgical intervention, and disease conditions such as diabetes, renal failure, infections, and autoimmune disorders (5, 6). Peripheral nerve injuries cause cognitive dysfunction via alteration of hippocampal neuroplasticity (7). Clinically, it mimics diabetic neuropathy and neurotrauma-associated cognitive dysfunction (8). Experimentally, peripheral nerve injuries by chronic constriction of the sciatic nerve injury (SNI) cause neuropathic pain along with an increase in the tumor necrosis factor-α (TNF-α) level in the hippocampus, dorsal root ganglia (DRG), and spinal dorsal horn (9). Further, the antagonist of the TNF receptor attenuates the neurobehavioural changes by reducing TNF-α synthesis in the brain regions (10). Sciatic nerve can cause a localized and central hypoxic environment with persistent vascular dysfunction and endoneurial hypoxia (11), whereas hypoxia post-conditioning protects against hypoxic/ischemic brain damage via upregulation of hypoxia-inducible factor 1 alpha (12). According to various literature reports, SNI causes cognitive impairment with alteration of synaptic plasticity and density in the hippocampus via enhancement of oxidative stress and inflammation (13).

Clinical manifestations have also revealed that SNI (sciatica) has a primary link to cognitive dysfunction with the presence of chronic pain rather than neuropathic pain itself (14). Multivariable analysis also revealed that lower back pain, waist pain, and especially sciatica pain are more likely to be associated with cognitive impairment (15). The area of the brain, the hippocampus, regulates cognitive functions in humans as well as animals. This area is also involved in the modulation of pain transmission via upregulation of BDNF levels (16, 17). Furthermore, the animal model of SNI-induced cognitive dysfunction closely resembles sciatica pain-related cognitive impairments in humans (18).

Numerous studies have revealed that natural nutritional compounds like chrysin and gallic acid protect brain function via regulation of blood-brain barrier functions (19). Folic acid is also called vitamin B9. It has various applications in food fortification, and it is also widely used as a dietary supplement. Additionally, folic acid helps prevent birth abnormalities caused by neural tube defects (20). It is considered an essential constituent in the dietary supplement for a pregnant woman. Furthermore, folic acid produces neuroprotective action via multiple cellular mechanisms such as restoration of AMPK activation, regulation of endothelial nitric oxide, potassium channels, and acetylcholinesterase activity (21-23). It also has curative properties in peripheral nerve injury (24). However, there is a lack of evidence of the effect of folic acid in the amelioration of cognitive dysfunction secondary to peripheral nerve injury. 

## 2. Objectives

The present study was designed to investigate the effects of folic acid on chronic constriction injury of the SNI-induced cognitive dysfunction in rats.

## 3. Methods

### 3.1. Animals Used

Disease-free male Wistar rats weighing 200 to 220 g were used in this study. They had access to standard laboratory feed and tap water ad libitum. Animals were kept under alternating light and dark cycles of 12 hours. Before the trial started, the rats were kept in the lab for a minimum of five days to allow them to acclimate. The Institutional Animal Ethics Committee (IAEC approval no.: 218/2017) approved the experimental design, and the animals were cared for in accordance with the Committee for the Purpose of Control and Supervision of Experiments on Animals' (CPCSEA) standard operating procedure, which is administered by the Ministry of Forest and Environment, Government of India.

### 3.2. Groups and Treatment Schedule

Six groups were used in this investigation. Six rats were employed in each group. In Group I, animals acted as the naive control. Group II animals acted as a sham control group. Group III animals acted as the SNI control. Groups IV and V animals received folic acid (10 and 20 mg/kg, p.o.; for 15 days), respectively (25). In Group VI, rats received donepezil (1 mg/kg) orally for 15 consecutive days (26). The drugs folic acid and donepezil were administered immediately after SNI surgery.

### 3.3. Induction of Cognitive Dysfunction via SNI

SNI was inflicted by chronic constriction injury as described by the method of Ren et al. (27). Silk threads were used for ligation of the sciatic nerve before the trifurcation branch, and each ligation was made 1 mm apart using the double-knot technique. Povidone ointment was applied at the site of the wound as post-operative care.

### 3.4. Assessment of Cognitive Function by the MWM Test

Spatial memory function was evaluated using the MWM test as described by Morris (28). In short, the MWM test apparatus was made up of a circular pool 150 cm in circumference and 45 cm in height filled with water to 30 cm. Each rat underwent four quadrant trials on each training day, i.e., day 1: Q1, Q2, Q3, and Q4 order; on day 2: Q2, Q3, Q4, and Q1 order; on day 3: Q3, Q4, Q1, and Q2 order; and on day 4: Q4, Q1, Q2, and Q3 order. The target quadrant, i.e., Q4, remained constant throughout the complete training period of this study. The animal's time to arrive at the hidden platform is known as the ELT, as the index of acquisition (learning) training. The cutoff time was set at 120 seconds. The rat was put in the pool and given 120 seconds to explore the water on the fifth day. The time spent in the target quadrant (TSTQ), i.e., Q4, was assessed as an index of retrieval of stored information.

### 3.5. Estimation of Biomarkers

According to the Ohkawa et al. method, the brain TBARS level was calculated (29). A DU 640B spectrophotometer (Beckman Coulter Inc., California, USA) was used to quantify the variations in absorbance as the optical density value at 532 nm. According to the Ohkawa method, the brain GSH level was calculated (30). A DU 640B spectrophotometer was used to quantify the variations in absorbance as the optical density value at 412 nm. According to Ellman et al., the method for calculating the brain AChE activity level was developed (31). A DU 640B spectrophotometer was used to quantify the variations in absorbance as the optical density value at 420 nm. According to Lowry et al., the method for calculating the brain total protein level was described (32). A DU 640B spectrophotometer was used to quantify the variations in absorbance as the optical density value at 750 nm.

### 3.6. Statistical Analysis

The standard deviation (SD) was used to examine the study's data. Two-way analysis of variance (ANOVA) was used to examine the behavioral test results. The post hoc test that was employed was the Bonferroni test. One-way ANOVA was utilized to evaluate the tissue biomarker data, and Tukey's multiple range test was employed as the post-hoc test. GraphPad Prism version 5.0 was used for all statistical analyses. 0.05 was chosen as the alpha value.

## 4. Results

### 4.1. Effect of Folic Acid on SNI-Induced Changes of Learning Behavior in the MWM Apparatus

**Figure 1.**
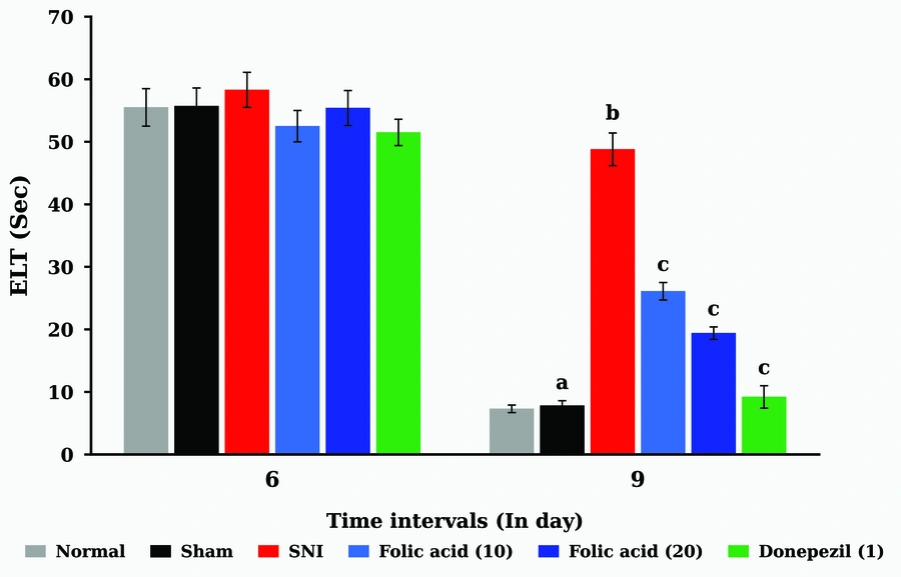
Effect of folic acid on sciatic nerve injury (SNI)-induced changes of ELT in the MWM test

The application of ligation of the SNI induces significant (P < 0.05) neuropathic pain associated with impairment of learning behavior (ELT) in contrast to the sham control group. When folic acid (10 and 20 mg/kg; p.o.) is administered, the learning behavior modifications brought on by SNI are considerably lessened than in the SNI control group (P < 0.05). The comparison medication, donepezil (1 mg/kg; p.o.), treatment group data showed comparable effects (P < 0.05). In the Figure 1 ‘a’ symbol indicates the reduction in the 9th day reading of ELT value of the sham control group when compared to the 6th day ELT value of the sham control group; the ‘b’ symbol indicates the increase in the 9th day reading of ELT value of the SNI group when compared to the 9th day reading of ELT value of the sham control group; and the ‘c’ symbol indicates the reduction in the 9th day reading of ELT value of folic acid and donepezil treatment groups when compared to the 9th day reading of ELT value of the SNI control group. Figure 1 displays the ELT modifications as shown below.

### 4.2. Effect of Folic Acid on Sciatic Nerve Injury-Induced Changes of Memory Retention in the MWM Apparatus

**Figure 2.**
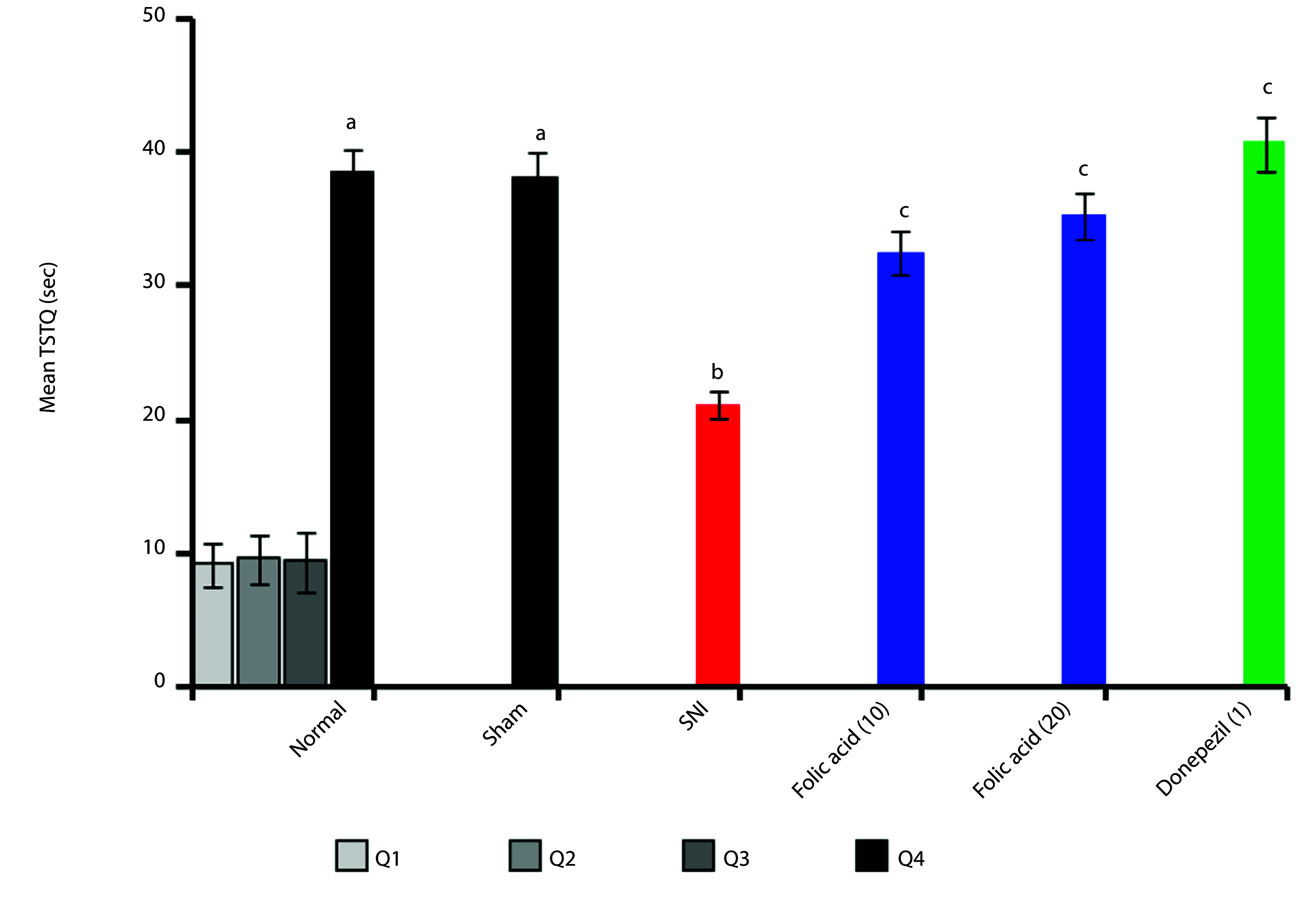
Effect of folic acid on sciatic nerve injury (SNI)-induced changes of TSTQ in the MWM test

The application of ligation of the SNI induces significant (P < 0.05) neuropathic pain-associated impairment of memory behavior (TSTQ) compared to the sham control group. Administration of folic acid (10 and 20 mg/kg; p.o.) significantly (P < 0.05) ameliorates the SNI-induced memory behavior changes compared to the SNI control group. The comparison medication, donepezil (1 mg/kg; p.o.), treatment group data showed comparable effects (P < 0.05). In the Figure 2 ‘a’ symbol indicates the increase in mean TSTQ reading as the Q4 value of the sham control group when compared to the Q1 value of the normal control group; ‘b’ symbol indicates the reduction in mean TSTQ reading as the Q4 value of the SNI control group when compared to the Q4 value of the sham control group; and ‘c’ symbol indicates the increase in mean TSTQ reading as the Q4 value of folic acid and donepezil treatment groups when compared to the Q4 value of the SNI control group. Figure 2 displays the TSTQ modifications as shown below.

### 4.3. Effect of Folic Acid on Biomarker Levels

The application of ligation of the SNI induces brain biochemical (TBARS, GSH, and AChE) changes when compared to the sham control group (P < 0.05). When folic acid (10 and 20 mg/kg; p.o.) is administered, the biomarker changes brought on by SNI are considerably lessened than in the SNI control group (P < 0.05). The comparison medication, donepezil (1 mg/kg; p.o.), treatment group data showed comparable effects (P < 0.05). Table 1 displays the biomarker modifications as shown below.

**Table 1.** Effect of Folic Acid on Biomarker Levels ^a^

Groups	TBARS (nM/mg of Protein)	GSH (μM/mg of Protein)	AChE (μM/mg of Protein/min)
**Normal**	18.4 ± 2.1	19.5 ± 1.2	3.5 ± 0.73
**Sham**	23.2 ± 1.9	20.1 ± 1.7	3.1 ± 0.46
**SNI**	58.2 ± 2.1 ^b^	8.4 ± 1.4 ^b^	15.7 ± 0.41 ^b^
**Folic acid (10)**	41.7 ± 1.7 ^c^	13.7 ± 0.6 ^c^	9.9 ± 0.59 ^c^
**Folic acid (20)**	26.8 ± 1.9 ^c^	17.3 ± 1.8 ^c^	6.2 ± 0.93 ^c^
**Donepezil (1)**	24.6 ± 2.2 ^c^	18.6 ± 2.1 ^c^	4.1 ± 0.68 ^c^

Abbreviations: AChE, acetylcholinesterase; GSH, reduced glutathione; SNI, sciatic nerve injury; TBARS, thiobarbituric acid reactive substances.

^a^ Dosage in mg/kg is indicated by the numbers in parentheses. Each group consisted of six rats, and the data were presented as mean ± SD.

^b^ P < 0.05 in comparison to a sham control group.

^c^ P < 0.05 in comparison to the SNI control group.

## 5. Discussion

In this research work, SNI showed impairment of spatial learning and memory functions in rats. SNI induced neuropathic pain along with impairment of spatial memory (13). The dysfunction of spatial memory is due to hippocampal neurodegeneration. The presynaptic neuronal boutons were reduced in the hippocampal CA1 region (33). Peripheral nerve injury leads to an elevated level of TNF-α in the hippocampus, cerebrospinal fluid, and plasma. Tumor necrosis factor-α is a proinflammatory marker, and it is associated with dysregulation of synaptic plasticity and memory impairment (4). One of the most well-established techniques for evaluating mice's capacity for learning and memory is the MWM (34). Normal control and sham control animals showed a significant decrease in ELT on day 9 and an increase in TSTQ compared to disease control animals. Furthermore, oral administration of folic acid (10 and 20 mg/kg) for ten days in a row attenuates SNI-induced cognitive impairments with a profile similar to that of a conventional nootropic agent, i.e., donepezil.

Further, folic acid treatment also significantly reduces brain AChE activity and brain oxidative stress levels when compared to SNI-operated animals. Our study showed that SNI-operated animals had reduced GSH levels and increased TBARS and AChE activity levels. Moreover, folic acid treatment decreases the SNI-induced changes in AChE, TBARS, and GSH levels, which indicates that folic acid possesses the ability to regulate peripheral nerve injury-induced alteration of brain biomarkers. The results are aligned with literature evidence, i.e., folic acid attenuates autoimmune disease progression with regulation of oxidative stress, inflammation, and DNA damage due to its pleiotropic functional properties, i.e., free radical scavenging, enhancement of antioxidant defense enzymes, inhibition of cytokine synthesis, and activation of cholinergic neurotransmission actions (35).

Folic acid's primary mechanism is to enhance conversion to tetrahydrofolate (THF) for the process of one-carbon transfers, which is needed in the synthesis of DNA and RNA, and the methylation process in amino acid metabolism (36, 37). Furthermore, it is essential for cell division, cell growth, and red blood cell formation (38). Hence, it plays a vital role in cardiovascular and neurovascular protective actions with reduction of free radicals, homocysteine (toxic metabolite), and inducible nitric oxide synthase (iNOS) against endothelial dysfunction (37, 39). Besides, folic acid also possesses neuroprotective action against peripheral neuropathy (40) and cognitive effects (41). It is also known to promote the repair process of peripheral nerve injury and neuropathic pain via regulating methionine cycle metabolism (24, 40).

**Figure 3.**
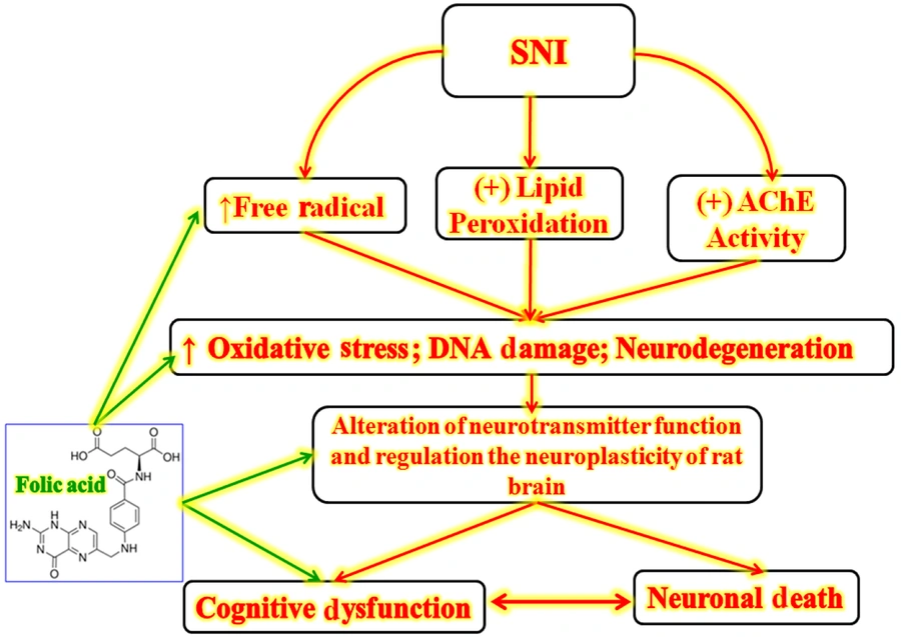
Salient findings of folic acid effect against the sciatic nerve injury (SNI)-induced cognitive impairment with molecular mechanisms

Furthermore, a conventional nootropic agent (i.e., donepezil) also protects neurons with a similar mechanism and enhances cognitive functions (42). Further, folic acid and donepezil reduce peripheral nerve injury-associated neuronal cholinergic neuron dysfunction (43, 44). The salient findings of the folic acid-associated molecular mechanism potentially ameliorate cognitive dysfunction against SNI (Figure 3). 

### 5.1. Conclusions

The short-term treatment of folic acid possesses neuroprotective and cognitive-improving actions via multiple pharmacological mechanisms. It is also evident from the results obtained in the various investigations. Therefore, folic acid may be used for peripheral nerve injury-induced cognitive dysfunction due to its potential pleiotropic actions. Hence, further extensive studies are required to explore the effect of folic acid and its molecular mechanisms for long-term neuroprotective actions against peripheral nerve injury.

### 5.2. Limitations of the Study

However, one of the notable limitations of this study is that it was carried out over a short treatment duration, i.e., 10 days, which may not fully capture the long-term neuroprotective effects of folic acid.

## Data Availability

The dataset used and analyzed in the current study is available upon request.
